# The Role of the Human Gut Microbiome in Inflammatory Bowel Disease and Radiation Enteropathy

**DOI:** 10.3390/microorganisms10081613

**Published:** 2022-08-09

**Authors:** Darren Fernandes, Jervoise Andreyev

**Affiliations:** 1 The Department of Gastroenterology, United Lincolnshire NHS Trust, Lincoln County Hospital, Lincoln LN2 5QY, UK; 2 The Biomedical Research Centre, Nottingham Digestive Diseases Centre, School of Medicine, University of Nottingham, Nottingham NG7 2RD, UK

**Keywords:** microbiome, inflammatory bowel disease, radiation, radiotherapy

## Abstract

The human gut microbiome plays a key role in regulating host physiology. In a stable state, both the microbiota and the gut work synergistically. The overall homeostasis of the intestinal flora can be affected by multiple factors, including disease states and the treatments given for those diseases. In this review, we examine the relatively well-characterised abnormalities that develop in the microbiome in idiopathic inflammatory bowel disease, and compare and contrast them to those that are found in radiation enteropathy. We discuss how these changes may exert their effects at a molecular level, and the possible role of manipulating the microbiome through the use of a variety of therapies to reduce the severity of the underlying condition.

## 1. Role of the Human Gut Microbiome

The human gut microbiome is made up of a collection of microorganisms, including bacteria, viruses, archaea, and protists [1]. It is a collection of over 10^14^ microorganisms, and plays a key role in regulating host physiology [2,3].

They assist in metabolising indigestible polysaccharides, producing vitamins that are required for the growth and maturation of intestinal epithelium and immune system; defend against attack by opportunistic pathogens [2]; and are instrumental in sustaining tissue homeostasis. In a “stable” state, both the microbiota and the gut work synergistically. Dysbiosis is a term that is used to describe an alteration that results in the gut microbiota composition being changed to one less harmonious, which can occur for a variety of reasons, including a change in diet, enteric infections, the use of antibiotics, or abdominal surgery [4].

Inflammatory bowel disease (IBD), which includes Crohn’s disease (CD) and ulcerative colitis (UC), is a significant burden to health. It is more prevalent in the Western world, especially in the United States and Europe [5]. However, recent analysis shows that it is now becoming a global health problem, with increasing incidence and prevalence in different parts of the world [5]. It may present in a number of ways, varying from presenting with a relatively mild disease phenotype to a very severe phenotype. It is not currently possible to predict the onset of IBD or the course of the disease in humans. This is because the aetiology is unknown, as are many of the underlying pathological processes [6]. However, IBD is thought to be multifactorial, where diet and environment trigger a dysregulated intestinal mucosal response to intestinal microbiota in genetically susceptible individuals [6].

In contrast, radiation enteropathy is a potentially more valuable human model of gastrointestinal (GI) inflammation and fibrosis. First, patients treated with radiotherapy (RT) for cancer often develop progressive changes that are initiated by the RT, and these changes can be followed over time. Secondly, patients receiving RT for pelvic tumours frequently complain of GI-related symptoms similar to those suffered by patients with IBD—including ulceration and bleeding, diarrhoea, steatorrhoea, haemorrhoids, nausea, and abdominal or anal pain [7]—which significantly affect their quality of life (QoL). These problems caused by RT are sometimes called “pelvic radiation disease” (PRD).

## 2. The Oxygen Hypothesis

There is a decrease in gut oxygen levels from the duodenum to the large intestine [8]. This is a consequence of the host being able to maintain a high oxygen concentration in the proximal small intestine [8], whilst also delivering nitrate to the distal end [9]. The microbial community composition of the small intestine is therefore dominated by facultatively anaerobic bacteria [10], whereas in the large intestine, there is predominantly obligately anaerobic bacteria as a consequence of limited oxygen and nitrate [11].

An imbalance between these obligate and facultative anaerobes is what characterises IBD dysbiosis [12]. The key factors influencing this imbalance are oxygen and reactive oxygen species (ROS) [13]. The “oxygen hypothesis” suggests there is an increased release of oxyhaemoglobin and ROS into the intestinal lumen due to chronic inflammation of the intestinal walls [14]. Radiation-induced dysbiosis has similarities to IBD, also exhibiting increased levels of ROS [15]. These changes create a microenvironment that favours facultative anaerobes [16,17]. As a consequence, the proportions of obligate anaerobes, such as *F. prausnitzii*, that release anti-inflammatory compounds are reduced, which leads to increased inflammation [18], creating a positive feedback loop that enhances the disease process [19]. 

## 3. IBD and the Gut Microbiome

Early studies on animal models have revealed that immune cells are unable to cause inflammation without the presence of intestinal bacteria, thereby indicating a role for the intestinal microbiome in the induction and/or maintenance of local inflammation and disease [20]. This is further supported by the finding that intestinal inflammation is most severe in parts of the bowel with larger bacterial populations [21]. Other studies have shown that specific patterns in microbiotic changes may be linked to the risk of IBD [21].

**Figure 1:**
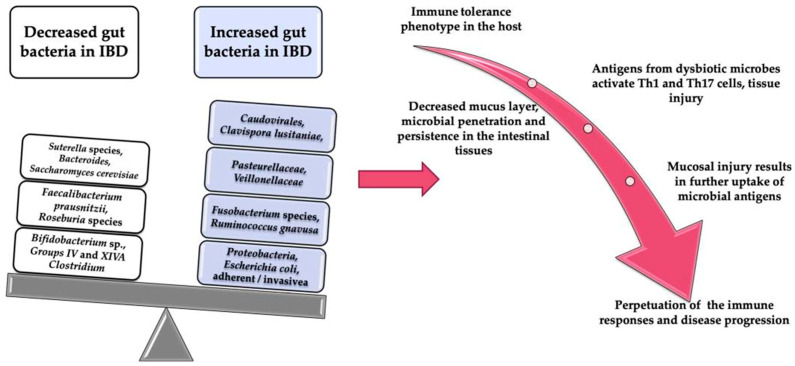
Altered gut bacteria implication in the pathogenesis of IBD. (Taken from Pavel et al., 2021 [38]).

The changes that have been described in the literature are shown in Table 1. The most consistent alterations detected in the gut microbiota of patients with IBD are a reduction in diversity, particularly of Firmicutes, compared to healthy individuals [22,23,24]. Increases in some species may promote inflammation, while reductions in others may do the same. These changes lead to a reduction in mucosal integrity either directly or by affecting colonic butyrate production, an important fuel for colonic epithelium, or by influencing cytokine production. The mechanisms via which these changes occur are further explained in Figure 1.

**Table 1:** Summarising the changes in bacterial population in IBD and the likely effect of the change.

Bacterial Species		Nature of Change (Increase/Decrease)	Change in UC or Crohn’s Disease	Likely Effect of the Change
*Proteobacteria*	*Escherichia* *Salmonella* *Legionellales*	Varies	Both	Pro-inflammatory if increased [25,26], anti-inflammatory if decreased [23,27]. In CD patients, intestinal permeability is increased due to adhesion-invasive *E. coli*, which leads to inflammation [28,29].
*Bacteroidetes*		Varies	Both	Pro-inflammatory if increased [25,26], anti-inflammatory if decreased [23,27].
*Firmicutes*	*Faecalibacterium prausnitzii*	Decreased	Both	Pro-inflammatory [30,31] and increased risk of post-operative occurrence in CD [32] due to reduction in short-chain fatty acids, especially butyrate [33]; this has an anti-inflammatory effect, provides energy for colonic epithelial cells, may strengthen epithelial barrier integrity, and plays a role in GI immune responses [34]. Recovery of population associated with maintenance of clinical remission in UC [32] due to production of interleukin (IL)-10 and inhibition of inflammatory cytokines, such as IL-12 and interferon-γ [35].
	*Roseburia inulinvorans*	Decreased	CD	Pro-inflammatory [30,31], higher genetic risk of IBD in healthy individuals with decreased levels [36].
	*Ruminococcus torques*	Decreased	CD	Pro-inflammatory [30,31]
	*Blautia faecis*	Decreased	CD	Pro-inflammatory [30,31]
	*Clostridium lavalense*	Decreased	CD	Pro-inflammatory [30,31]
	*Erysipelotrichales* *Clostridiales*	Decreased	CD	Pro-inflammatory due to reduction in butyrate production [28,29,36,37].
	*Veillonellaceae*	Increased	CD	Pro-inflammatory [36]
*Enterobacteriaceae* *Pasteurellaceae* *Fusobacteriaceae*		Increased	CD	Pro-inflammatory [36]

## 4. Studies Assessing the Microbiome of Twins with IBD

A further insight into the role of the gut microbiome in the pathogenesis of IBD is through studies looking at the development of IBD in twins. Previous studies exploring this have reported differences in the gut microbiome composition in IBD-affected twins compared with their healthy co-twins [39,40,41,42,43] (see Table 2).

However, these studies were performed in small numbers, no more than 10, of IBD-discordant or concordant twin pairs [40,41,42,43], and either did not include an unrelated matched healthy control group [39,43], or only included a small non-matched control group [40,41,42]. In addition, they were based on 16S rRNA sequencing [39,41,42,43], which does not assess microbial functional pathways.

A more recent study by Brand et al. [44] showed no significant differences in the relative abundance of species and pathways between healthy co-twins and their IBD-twins. However, they found an overlap in species, between healthy co-twins and IBD-twins and healthy co-twins and unrelated patients with IBD, respectively. Many of these shared species have previously been associated with IBD, such as *Escherichia unclassified*. The gut microbiome may therefore display IBD-like signatures that precede the onset of IBD [44]. However, longitudinal follow-up studies are needed to infer a causal relationship [44].

## 5. The Effects of Radiotherapy on the Gut Microbiome

Both curative and palliative cancer patients may receive RT, in combination with chemotherapy, as part of their cancer care [45]. Approximately 50% of all cancer patients receive RT [46], with 90% of those receiving pelvic RT developing a permanent change in their bowel habits [47]. Despite the well-established benefits of RT in oncology, RT-induced toxicities may detract from this, significantly impairing the QoL of patients. Radiation enteritis can be either acute or chronic. The chronic form, more correctly called radiation enteropathy, usually develops between 3 months to many decades after treatment [47], and occurs in approximately 5% to 55% of patients treated with pelvic RT [48]. Severe symptoms arising from chronic radiation enteropathy not only affect cancer patient’s QoL, but also add to the cost of medical treatment by increasing the use of medication for symptom relief, prolonging hospital stays, and temporarily or permanently stopping cancer treatment [49,50].

There is a close relationship between gut microbiota dysbiosis and intestinal injury after RT. This is summarised in Table 3.

## 6. The Human Gut Virome

The human gut virome contains eukaryotic viruses, prokaryotic viruses, and phages, known as bacteriophages and bacterial viruses (the phageome) [68]. According to the Global Virome Database, phages make up 97.7% of the gut virome, with 2.1% being eukaryotic viruses and 0.1% being prokaryotic viruses [69]. Cross-kingdom interactions between phages and bacteria, and between viruses/phages and the host immune system, underlie the function of the human gut virome in health and disease [70]. The virome population can affect its host in the following ways [70]:

- Eukaryotic viruses that infect human cells trigger immune responses, which can then lead to disease.
- Phages can affect the host indirectly via modulation of bacterial composition and bacterial fitness.

Potential activators of chronic inflammation can be released when enteric eukaryotic viruses and bacteriophages kill host cells [71]. The resulting dysbiosis of the enteral system is one of the key factors in the pathogenesis of I BD [72]. Several studies have delineated the events that can occur in inflammatory bowel disease (Table 4). There are no studies yet in patients treated with radiotherapy. As a result of significant improvements in sequencing technology in recent times, the diversity of the enteric human virome is being increasingly revealed, leading to new ways of targeting the gut microbiota to prevent or treat disease [71].

## 7. How the Microbiome Acts as the Guardian of the Gut from Radiation: Parallels with IBD

It has been established that toll-like receptors (TLRs) act as the centre of immune responses to microbes in the gut [83]. TLRs are a group of proteins that are expressed by a number of immune cells, including macrophages, neutrophils, dendritic cells, and epithelial cells [4]. They recognise pathogen-associated molecular patterns (PAMPs), which are highly conserved structures of microbes [84]. Upon activation, TLRs induce a number of inflammatory cytokines by mediating the phosphorylation of IκB to activate NF-κB [83]. It also regulates the maturation of dendritic cells (DCs), and the proliferation and differentiation of Th1 and Th2 T cells [83].

A proliferation-inducing ligand (APRIL) and thymic stromal lymphopoietin (TSLP) are cytokines that are expressed following stimulation of intestinal epithelial cells (IECs), which promote class switch recombination (CSR) of IgM and IgA1 to protease-resistant IgA2 [85]. IgA2 stops invasion by bacteria by binding them to the apical surface of IECs [85]. The production of trefoil factor 3 (TFF3) is also increased following the activation of TLR2, which promotes the repair of gaps in the epithelial monolayer [85]. Microbicidal peptides and lectins, such as α-defensins and regenerating islet-derived protein 3γ (REG3γ), are released by Paneth cells due to TLR stimulation [85].

TLR signalling facilitates the optimal functioning of the immune mechanisms within a healthy host by protecting barrier integrity and maintaining commensal composition and tolerance. However, dysfunctional TLR signalling in susceptible individuals may impair commensal–mucosal homeostasis, thus leading to a worsening of tissue injury and ultimately to chronic inflammation in IBD [86] (see Table 5).

Similarly, as in IBD, the gut microbiome may prevent injury induced by radiation through the activation of TLRs [4]. Entolimod, a TLR5 ligand, has previously been shown to decrease the rate of apoptosis of intestinal crypt cells, as well as cells within the lamina propria in mice and primates, when given as a pre-irradiation injection [102]. In another study in mice, pre-treatment with a TLR9 ligand reduced small bowel radiation injury through a MyD88-dependent signalling pathway [103]. Bacterial flagellin and CpG (cytidine–phosphate–guanosine) DNA, which are TLR5 and TLR9 ligands, are found in bacteria and viruses, respectively [4].

Additionally, the use of lipopolysaccharide, a membrane component of Gram-negative bacteria, before radiation provides protection to intestinal crypts via the induction of cyclooxygenase-2 and the production of prostaglandins [104]. The release of tumour necrosis factor (TNF)-α, which occurs due to the stimulation of TLR4-expressing cells by lipopolysaccharide, also results in increased production of prostaglandins and decreased radiation-induced apoptosis of epithelial stem cells [105]. TLR may also exert its protective effects against radiation through the activation of nuclear factor-kappa B (NF-κB) signalling [106], which is required for defending the gut against radiation-induced apoptosis. NF-κB activation also moderates the radioprotective effects of lipopolysaccharide [107], suggesting that TLRs have an effect on the intestinal response to radiation-induced epithelial damage through the NF-κB pathway [108].

In contrast, radiation damage of the bowel can be worsened through the activation of TLR3, and possibly TLR4. Injection with the TLR3 ligand Poly I:C, found naturally in viruses, resulted in more severe GI symptoms after whole body irradiation [4]. In addition, TLR3 knockout mice appeared more radioresistant by having less apoptotic intestinal epithelial cells, and also a larger proportion of radiation surviving crypts. Another study using knockout mice revealed that pre-treatment with the TLR4 antagonist, C34, reduced radiation-induced cell damage and death [109].

These compounds may provide the bowel with radioprotection through their effect on the systemic immune system. Furthermore, it is has been established that germ-free mice are more able to withstand radiation-induced bowel injury than conventional mice colonised with the microbiome [110,111]. These findings suggest that a key factor in the development of radiation enteropathy could be the gut microbiota, thus allowing the possibility to prevent or treat radiation enteropathy by manipulating its composition.

## 8. Treatment in IBD

There are several ways of regulating the gut microbiota during therapy. One example is the use of mitochondria-associated membrane (MAM) proteins when there is a reduction in anti-inflammatory bacteria; these are anti-inflammatory molecules produced by *F. prausnitzii* [112]. In cases such as this, probiotics, prebiotics, synbiotics, and antibiotics can be utilised to replenish anti-inflammatory bacteria and their substrates. Another way is to target the inflammatory bacteria with antibiotics or phage therapy. Faecal microbiota transplantation (FMT) can also be used to reset the whole microbiome. Research has shown the therapeutic benefits of the microbiota; for example, altered organisms, whose purposes are to release anti-inflammatory cytokines or other molecules, can be delivered straight to the area of inflammation [113]. 

## 9. Probiotics

Probiotics are microorganisms that are able to withstand the acidic environment of the stomach. A number of ways in which probiotics act have been proposed [114,115,116,117]:

- Triggering a rise in anti-inflammatory cytokines (IL-10, transforming growth factor beta (TGF β)).
- Release of antimicrobial products and halting of bacterial development.
- Stimulating the immune response.
- Enhancing epithelial barrier function.
- Stopping T-cell generation.

In order for the microorganisms to be classed as probiotic agents, they must have the following criteria [38]:

- To be able to withstand the acid secretions of the stomach, gallbladder, and pancreas, thus remaining viable when they reach the small and large intestines.
- To remain functionable during transfer and storing.
- Not to have any adverse effect on normal tissue structures.
- To benefit the host.
- To stick to intestinal epithelial cells.
- To stabilise the intestinal microbiota.
- To secrete antimicrobial products.

The evidence that probiotic treatment is effective in IBD is outlined in Table 6. Effects may differ slightly in UC and CD, and different strains of probiotics have been trialled under the two conditions. Concomitant use of multiple strains in patients seems to have better outcomes than the use of a single microorganism. The optimal doses have not been determined yet, and a number of studies use doses above what is recommended, while other studies do not state the dose given [38].

## 10. Prebiotics

Prebiotics are indigestible carbohydrates that are broken down by select bacteria in the intestine, resulting in their growth and providing benefit to their host [133].

Prebiotics are comprised of inulin, fructo-oligosaccharide (FOS), galacto-oligosaccharide (GOS), and lactulose, which occur in higher levels in healthy populations of commensal *Lactobacillus* and/or *Bifidobacterium* spp. [134]. Prebiotic use in IBD works in a variety of ways, including the selective growth of native bacteria within the intestinal microbiota, and the enhanced production of SCFAs (i.e., acetate, butyrate, and propionate) [135].

There have been several studies in animal colitis models and IBD patients demonstrating the potential benefits of prebiotic use [136,137,138,139,140]. Both inulin and FOS reduced prolonged intestinal inflammation in HLA-B27 transgenic rats through regulation of the gut microbiota, and by increasing the availability of probiotics *Bacteroides–Prevotella–Porphyromonas* and *Bifidobacteria* [141]. Moreover, it was found, in IBD models, that these agents play a significant role in reducing 2, 4, 6-trinitrobenzene sulfonic acid (TNBS)-induced colitis by increasing the abundance of probiotics (*Lactobacillus* and *Bifidobacterium*) and the production of SCFAs [142]. Although the effects of prebiotics are promising, studies of their use in IBD remain limited and controversial [136]. In short, it cannot be definitively concluded that they improve IBD symptoms [143], and so further research is required to confirm their potential benefits.

## 11. Synbiotics

Synbiotics are a mixture of probiotics and prebiotics [144,145], and require the prebiotic compound to selectively favour the probiotic organism. They were developed to improve the survival of probiotics when passing through the upper GI tract. The purpose of a synbiotic is, therefore, to facilitate the delivery of a probiotic to the colon and to augment the growth of probiotic strains [146]. Furrie et al. [147] found that there were reduced microscopic inflammatory lesions of the rectal mucosa, and also lower levels of pro-inflammatory cytokines, such as TNF-α and IL-1β, following use of a synbiotic consisting of *B. longum* and oligofructose-enriched inulin in UC patients. However, a study in children with IBD by Hansen et al. [148] did not confirm this finding. Chermesh et al. [149] also found that there was no benefit to post-operative recurrence in 30 patients with CD that were treated with a mixture of four probiotic species and four prebiotics. Nonetheless, there may well be a beneficial effect of using a variety of selected synbiotics on the intestinal mucosa in IBD, but further investigation into their use is required [144].

## 12. Faecal Microbial Transplantation

FMT is a therapy in which faecal matter from a healthy donor is placed into the GI tract of a patient with a chronic condition so that it can be treated by restoring the normal intestinal microbiome [150]. Currently, FMT is frequently used in the management of recurrent *Clostridioides difficile* infections [151]. It has also been shown to be useful in the treatment of patients with IBD, with one meta-analysis showing an effectiveness of 21% in UC and 30% in CD patients [152]. In another study, FMT use was found to be beneficial in 20.9% of patients with mild to moderate IBD, and 32.3% of those with moderate to severe IBD [153]. This suggests that FMT may be more efficacious in those with moderate to severe IBD, and could be considered as an alternative rescue therapy for refractory disease. However, there are substantial differences between recently conducted studies due to variances in transplantation methods and routes, as well as through the use of fresh or frozen faeces and the selection criteria of donors.

The mechanisms through which FMT benefits patients are thought to be associated with the alteration of the intestinal microbiota, with an increase in diversity and the composition shifting towards that of the donor profile [154]. Paramsothy et al. [153] also revealed a rise in the diversity of intestinal flora and an alteration in its composition following FMT [155]. In patients who benefitted from FMT, their faecal and colon samples contained higher levels of *Eubacterium hallii*, and clinical remission positively correlated with donor stools that contained *Bacteroides* species [156]. However, there was no response to FMT if the donor stool contained *Streptococcus* species [157]. Further studies are therefore required in order to ascertain the best way of selecting donors, so that FMT can exert its most beneficial effect on the microbiome changes that occur in IBD patients.

## 13. Antibiotics

Antibiotics can affect the clinical course of IBD by decreasing bacterial concentrations in the lumen, and also by changing the intestinal microbiota composition to a more advantageous one [117,158]. Table 7 and Table 8 detail the studies that have been performed assessing the use of antibiotics in both active CD and active UC. It is important to note that there is less data concerning the treatment of UC with antibiotics, which consists of studies that contain a small number of patients and a lack of well-designed, placebo-controlled trials [159].

## 14. Diet

Fats, proteins, carbohydrates, and fibres can all have an impact on the onset of IBD, with a Western diet associated with an increase risk [33]. However, there are no studies assessing similar uses in patients post RT. Therefore, we will only focus on the benefits of following specific diets in terms of the microbiome in IBD (Table 9).

## 15. Faecal Virome Transplantation

Faecal virome transplantation (FVT) is a refined method of FMT that removes faecal bacteria, thereby decreasing the risk of bacterial infection associated with FMT [190]. Several studies in non-IBD patients have shown much promise for its use (see Table 10).

Although the efficacy of FVT has been established in non-IBD patients, we need to remain cautious as pathogenic eukaryotic viruses can be co-transferred along with phages, and thus pose potential health concerns [195]. This is particularly important in IBD patients and those treated for cancer, who may be immuno-compromised. Nonetheless, FVT presents a very useful method for treating microbiome-dysbiosis-related disease, and so further investigation into its efficacy and safety in this patient group should be performed.

## 16. Phage Therapy

Phage therapy is a process whereby virulent phages are given directly to the patient with the purpose of lysing the bacterial pathogen [196], and so represents a method of restoring intestinal eubiosis.

Some advantages linked to phage therapy include [197]:

- An ability to increase their number where their host is present.
- Being highly specific and infecting only a few bacterial strains.
- Remaining in an environment only when their hosts are present.
- Able to modify themselves in relation to evolving bacteria, allowing them to remain capable of infecting and lysing the bacteria.

There is currently limited research into the use of phage therapy in the management of IBD, and none with regards to treating radiation enteropathy. Currently, the use of phage therapy in IBD has been mainly targeted at adherent invasive *Escherichia coli* (AIEC), which has been shown to be more prevalent in CD patients [198], and in maintaining intestinal inflammation in IBD [199,200,201]. Studies have shown the potential efficacy of phage therapy against AIEC (see Table 11).

Although these recent studies on phage therapy are promising, further work is needed to learn more about their use and safety.

## 17. Therapeutic Options for Radiation-Induced Intestinal Injury

Radiation damage to the GI tract can be a severe complication that can contribute to multiple organ failure [206]. It has also been shown in many experimental models that multiple organ failure may be the result of excessive inflammatory responses following intestinal injury [207]. Therefore, targeting early intestinal changes that occur after radiation exposure may help to prevent or reduce radiation syndrome [208]. As used in IBD, the gut microbiota and its metabolites can be effective treatments for radiation-induced intestinal injury.

## 18. Probiotics

Probiotics were found, as early as 1988, to be useful treatments for GI symptoms occurring post RT (see Table 12).

These findings share parallels with the use of probiotics in IBD. Therefore, their use as mitigators against radiation toxicity is potentially very exciting, and so it is necessary that further research is conducted with regards to how best to improve the formulation, administration and absorption of these products.

## 19. Prebiotics

Prebiotics have also been shown to play an important role in immune regulation and have anti-tumour properties. In a randomised trial, Garcia-Peris et al. [215] found that SCFA-producing bacteria, such as *Roseburia,* were increased in pelvic RT patients who were given a mixture containing inulin and that this reduced the severity of diarrhoea [208]. However, studies assessing the use of prebiotics in patients receiving RT are limited and further work needs to be conducted to explore their potential benefit in reducing the risk of radiation enteritis.

## 20. Faecal Microbial Transplantation

It has been reported that FMT is safe and effective in patients with radiation enteropathy, improving intestinal symptoms and mucosal injury for a certain amount of time [216]. It can also be used to improve the prognosis of cancer patients after RT by ameliorating the toxicity that is caused as a result of radiation damage [217]. Recently, it has also been shown to be a successful treatment in immunotherapy-induced colitis [218]. Potential adverse effects that can occur as a result of transmission from the donor’s faeces may be avoided through careful selection of healthy donors. The standardisation of this therapy and the normalisation of its use are, therefore, two key factors in the usage of this treatment that are required to meet the needs of patients [219]. Additionally, similar to IBD, further studies are required to understand how best to target the microbiome in order to implement the changes needed to alleviate symptoms.

## 21. Antibiotics

Antibiotics have been shown to be beneficial in the restoration of gut microbes in irradiated mice. It has been reported that an antibiotic cocktail and metronidazole pre-treatment results in less severe intestinal inflammation, which occurs due to a reduced level of lipopolysaccharide (LPS) in the ileum and inhibition of TLR4/MyD88/NF-κB signalling [26]. In addition, antibiotic pre-treatment regulates macrophage polarisation in the ileum, and downregulates the expression of TGF-β1, phosphorylated Smad-3, and α-SMA, leading to reduced intestinal fibrosis [26,217]. These results provide evidence that antibiotic pre-treatment can be an effective means of easing gut microbial dysbiosis and intestinal injury caused by RT. It will therefore be important to further our understanding of the pathogenesis of radiation enteritis in humans, and the role antibiotics play in alleviating it.

## 22. Conclusions and Future

A number of processes can disturb the intestinal flora, as well as conditions, such as IBD and cancer, which alter the health status of the host and, thus, affect the overall homeostasis of intestinal flora. It is crucial that future studies are carried out that use healthy people as a control group, and that assess bacterial function as well as numbers.

This will allow for comparison and may lead to the revelation of bacterial genera that are altered in radiation enteritis. With regards to treatment, the microbiome of those with intestinal damage from radiation can be targeted so that it can be changed to a more healthy composition [220]. Our review has shown the many ways in which this is already being conducted in patients with IBD, and how it can possibly be performed on patients with radiation enteritis. The microbiota are the guardians of our gut, and we should use them to our benefit; this will require the development of considerable collaboration across medical and scientific disciplines, which currently rarely meet.

## Figures and Tables

**Table 2:** Summarising the changes in bacterial population in twin studies.

Phylum	Genus	Nature of Change
*Proteobacteria*	*Aeromonadaceae*	Increased in ICD [39]
	*Enterobacteriaceae*	Increased in ICD [39]
	*Escherichia*	Increased in UC [42] and ICD [43], decreased in CCD [43]
*Bacteroidetes*	*Prevotellaceae*	Decreased in ICD and CCD [39]
	*Bacteroidales*	
	*Bacteroides uniformis*	Decreased in CD [41]
	*Bacteroides ovatus*	Increased in CD [41]
	*Bacteroides vulgatus*	Increased in CD [41]
*Firmicutes*	*Ruminococcaceae*	Decreased in ICD [39]
		Increased in CCD [39]
	*Peptococcaceae*	Decreased in ICD [39]
	*Un_Clostridiales*	Decreased in ICD [39]
	*Lactobacillaceae*	Increased in ICD [39]
	*Faecalibacterium Roseburia*	Increased in ICD [40]
	*Coprococcus*	Increased in ICD [40]
	*Dialaster*	Increased in ICD [40]
	*Faecalibacterium prausnitzii*	Decreased in ICD [43]
*Fusobacteria*	*Fusobacteriaceae*	Increased in ICD [39]
		Decreased in CCD [39]
*Tenericutes*	*Anaeroplasmataceae*	Decreased in ICD [39]
		Increased in CCD [39]
*Actinobacteria*	*Rhodococcus*	Increased in UC

ICD = Intestinal Crohn’s disease, CCD = colonic Crohn’s disease, UC = ulcerative colitis.

**Table 3:** Detailing the observed changes in microbiota following exposure to radiation.

Study	Observed Change in Microbiota and Potential Causative Mechanisms in Inflammatory Response of the Gut
**Reis-Ferreira et al., 2019 [51].**	There is a link between radiation enteropathy (RE) and higher *Clostridium IV*, *Roseburia*, and *Phascolarctobacterium* counts. In addition, there was a reduction in intestinal mucosal cytokines associated with intestinal flora regulation and intestinal wall maintenance.
**Wang et al., 2019 [52].**	Richer number of *Proteobacteria*, *Gammaproteobacteria*, *Virgibacillus*, and *Alcanivorax*, but less *Bacteroides*, in patients with mild enteritis. RE-derived flora capable of initiating epithelial inflammation and barrier dysfunction, and enhancing the expression of TNF-α and IL-1β.
**Gerassy-Vainberg et al., 2018 [53].**	Rectal radiation induces dysbiosis, which is in part mediated by IL-1β; this results in an increased susceptibility to radiation and inflammation.
**Fernandes et al., 2021 [1].** **Bennett and Eley, 1993 [54].**	Increased abundance of *Proteobacteria* with decreased abundance of *Faecalibacterium* following exposure to ionising radiation [1]. In addition, there was also an increased relative abundance of bacteria belonging to the *Fusobacteria* phylum, which are known to be associated with an extensive spectrum of infections [54].
**Cuzzolin et al., 1992 [55]; Sajjadieh et al., 2012 [56]; Garcia-Peris et al., 2012 [57]; Yamanouchi et al., 2019 [58]; Yi et al., 2021 [59].**	*Bifidobacterium* and *Lactobacillus* genera display probiotic effects, and have been used in the management of GI conditions [56,57,58]. Two studies reported decreases in abundances of the genera *Bifidobacterium* and *Lactobacillus*, whilst another reported a decrease in *Lactobacilli* (aerobic and anaerobic) in subjects exposed to radiation [55,56,57]. Conversely, Yi Y et al. [59] reported an increase in *Lactobacillus*.
**Wang et al., 2015 [60]; Sahly et al., 2019 [61]; Wang et al., 2019 [52]; Yi et al., 2021 [59].**	*Bacteroides* is one of the most abundant genera in the human gut, and its members are vital in maintaining the stability of a healthy gut ecosystem [62]. They play an important role in the hydrolysis and fermentation of exogenous fibre and endogenous mucins, both in the deconjugation of bile acids and in the production of acetic and lactic acids [63,64]. Additionally, they play a part in stimulating the immune system, by augmenting the production of IL-2 by macrophages and B cells [56,65]. They have been found to be beneficial to the gut when present in other locations, but can cause significant infections [65]. The analysed studies reported mixed results: increases in relative abundance in two studies [60,61]; decreases in two other studies [52,59]. These studies were limited due to small sample sizes, not having a healthy control group, and for including patients on medications known to disrupt the gut microbiota [66,67], thus, making it more difficult to fully isolate the unique effect of radiation.

**Table 4:** Changes in the Hunan Gut Virome of Patients with IBD.

Study	Findings
Lepage et al., 2008 [73]	Biopsies of colonic mucosa of CD patients found that CD patients possessed significantly more virus-like particles (VLPs) than healthy individuals.
Wagner et al., 2013 [74]	Higher abundance of phages in paediatric CD patients compared to controls. *Bacteroides* phage B10-8 and phage B124-14 represented the largest proportion of sequences. Finally, the *Mycobacterium* phage composition in ileum tissue samples of CD patients was different compared to controls.
Perez-Brocal et al., 2015 [75]	Phages were three times more abundant in faeces than in colonic biopsies, and the disease status of individuals was more accurately reflected by the bacterial rather than the viral communities. Moreover, a number of viral biomarkers that are associated only with CD disease were identified. Finally, they found that there was a rise, in CD patients, in phages infecting bacterial orders *Alteromonadales* and *Clostridiales*, including bacterial species *Clostridium acetobutylicum* and the *Retroviridae* family.
Wang et al., 2015 [76]	Increased viral sequences in CD and difference in the abundance and diversity within the virome between CD and the control group.
Norman et al., 2015 [77]	Higher viral richness and *Caudovirales* growth in CD and UC patients, reduced bacterial richness and diversity in CD and UC, and a negative association between *Caudovirales* and prevalent bacterial taxa in CD.
Zuo et al., 2019 [78]	Larger number of *Caudovirales* phages, but reduced diversity, richness, and uniformity of mucosa *Caudovirales* in UC patients compared with healthy controls. In addition, there was a higher abundance of *Escherichia* and *Enterobacteria* phages in the mucosa of UC patients than in heathy controls.
Clooney et al., 2019 [79]	Showed that a healthy core of virulent phages is substituted by temperate phages in CD patients.
Fernandes et al., 2019 [80]	Paediatric IBD subjects had a greater relative abundance of *Caudovirales* to *Microviridae* phages compared to controls. The *Caudovirales* phages were also more abundant in CD than UC, but not controls. The richness of viral strains in *Microviridae*, but not *Caudovirales*, was increased in controls compared to CD but not UC.
Yan et al., 2020 [81]	Paediatric CD patients in a virome sequencing study showed higher diversity between patients, and low variation within patients, of wash samples taken from the proximal and distal colon.
Liang et al., 2020 [82]	No significant difference in the total number of VLPs between very early onset IBD, defined as the occurring before the age of 6 years, and healthy controls. However, the very early onset IBD subjects exhibited a higher ratio of *Caudovirales* vs. *Microviridae* compared to healthy controls.

**Table 5:** Role of TLRs in IBD. (Adapted from Lu et al., 2018 [83]).

TLRs	Role in IBD
TLR1/2	Stops chronic inflammation [87,88]
TLR2/6	Stimulates colitis [89,90]
	Dampens down the immune response [91]
TLR3	Assists in protective immunity under an inflammatory environment [92]
TLR4	Leads to a breakdown of intestinal tissue and ulceration [93,94,95]
	Has a defensive role [96]
TLR5	Inhibits diseases that can occur due to intestinal inflammation [97]
TLR7	Provides ability to fight of infection under inflammatory conditions [92]
TLR8	Stimulates inflammation of mucosa [98]
TLR9	Has a defensive role [99,100,101]

**Table 6:** Probiotics used in IBD.

	Probiotic	Effect
**Crohn’s Disease**	*Saccharomyces boulardii*	Reduced recurrence rates when combined with 5-ASA treatment [118].
		Reduces intestinal permeability and secondary bacterial translocation, as well as demonstrating an immunomodulatory effect by causing a rise in plasma levels of IL-10 and intestinal IgA secretion [119].
	Synergy 1 (containing *Bifidobacterium longum*, oligofructose, and inulin)	TNF-α, a pro-inflammatory biomarker in the intestinal mucosa, was reduced, as was disease activity, after 6 months of treatment. This was also found using histological indices [120].
	VSL#3 (containing *Bifidobacterium infantis*, *Bifidobacterium breve*, *Bifidobacterium longum*, *Streptococcus thermophilus*, *Lactobacillus paracasei*, *Lactobacillus acidophilus*, *Lactobacillus recarurus*)	Patients given VSL#3 immediately after surgery had reduced levels of Il-8 and IL-1b, which are pro-inflammatory cytokines, and also had lower rates of disease recurrence compared to those given treatment 90 days post-surgery [121].
**Ulcerative Colitis**	Combination of *Saccharomices boulardii* and VSL#3 with conventional therapy	No significant improvement in the remission rates of the disease, but found to be beneficial in decreasing disease activity [122].
	Combining VSL#3 with standard therapy	Endoscopic healing of colonic mucosa and a decrease in ulcerative colitis disease activity index (UCDAI) score by more than 50% after 12 weeks of treatment [123].
	VSL#3	The use of VSL#3 amongst children with UC in the induction and maintenance of remission is effective when either used alongside steroids and 5-ASA treatment [124], or when used alone [125].
	Bifidobacteria-fermented milk (a combination of *Bifidobacterium* strains and *Lactobacillus acidophilus*)	Improved endoscopic and histological scores in patients with UC [126].
	*Escherichia coli* Nissle *1917* *Bifidobacterium breve strain Yakult* *Bifidobacterium breve* *Saccharomyces boulardii*	Showed to have a comparable effect with 5-ASA in maintaining remission when used in patients with mild to moderate UC [127,128,129,130].
	*Saccharomyces boulardii*	Clinical remission of UC was maintained with 400 mg rifaximin and 500 mg *Saccharomyces boulardii* after 3 months of use. This treatment regimen may be helpful in preventing early relapses in UC [131], which is an important therapeutic target in the management of patients with IBD [132].

**Table 7:** Detailing studies assessing the efficacy of antibiotics in active Crohn’s disease.

Antibiotic	Study	Findings
Various combinations (including ciprofloxacin, metronidazole, rifaximin, clarithromycin)	Khan et al., 2011 [160]	Antibiotics were better at inducing remission of active CD compared to placebo.
	Wang et al., 2012 [161]	56.1% (214/429) of patients treated with antibiotics showed a response compared to 37.9% (153/403) of patients given the placebo.
	Su et al., 2015 [162]	The combined relative risk (RR) for clinical remission or response in patients with CD was 1.33.
Ciprofloxacin	Arnold et al., 2002 [163]	There were significantly lower disease activity scores in 47 patients with moderately active resistant disease who had been treated with a twice daily regime of ciprofloxacin 500 mg compared to those who received placebo only.
	Steinhart et al., 2002 [164]	Ciprofloxacin treatment was found to be more beneficial for those who had active disease and colonic involvement.
	Su et al., 2015. [162]	There was a similar clinical response rate between the ciprofloxacin and placebo group.
Metronidazole	Sutherland et al., 1991 [165]	There was minimal benefit of Metronidazole use in active CD, with a decrease in disease activity index but no difference in the rate of remission.
Combination of Ciprofloxacin and Metronidazole	Prantera et al., 1996 [166]	Although not statistically significant, the steroid group contained a higher number of patients in clinical remission.
	Steinhart et al., 2002 [164]	No difference in remission rates.
Rifaximin	Prantera et al., 1996 [166]	402 patients with CD received 12 weeks of treatment with extended release rifaximin; 62% of those given Rifaximin 800 mg were in remission compared with 43% who received the placebo.
	Khan et al., 2011 [160]	Able to induce remission, and led to a decreased risk of persisting active disease compared to the placebo.
	Jigaranu et al., 2014 [167]	All patients receiving Rifaximin 800 mg twice daily for 12 weeks achieved remission compared to 84% in the placebo group.

**Table 8:** Detailing studies assessing the efficacy of antibiotics in active ulcerative colitis.

Metronidazole	Chapman et al., 1986 [168]	In this RCT, 39 patients were given either metronidazole with steroids or placebo with steroids for 5 days. There was no significant difference between either treatment group.
	Gilat et al., 1987 [169]	From a prospective RCT, it was found that 1.35 g/day of oral metronidazole was ineffective in managing an attack flare of non-severe UC compared to 4.5 g/day of sulfasalazine.
	Mantzaris et al., 1997 [170]; Mantzaris et al., 2001 [171]	Two RCTs found no significant difference in clinical improvement when assessing intravenous or oral treatment with metronidazole alongside steroids for 2 weeks in patients with mild to severe UC.
Metronidazole/Tobramycin	Burke et al., 1990 [172]	In 84 patients with an acute flare of UC that were randomised to receive either oral tobramycin or placebo alongside steroid therapy for 1 week, 74% of those given tobramycin achieved complete symptomatic remission, compared with 43% in the placebo group. There were also better histological scores at the study endpoint in the tobramycin group.
	Mantzaris et al., 1994 [173]	In 39 patients with severe UC received either metronidazole and tobramycin or placebo in addition to total parenteral nutrition (TPN), IV hydrocortisone, and hydrocortisone enemas. In total, 66% of patients given antibiotics, and 65% of those that took the placebo, showed considerable improvement.
Ciprofloxacin	Turunen et al., 1998 [174]	This study revealed that 6 months of ciprofloxacin treatment compared to placebo, in addition to steroids, in 83 patients known to be poor responders to conventional therapy, resulted in a lower rate of treatment failure; 21% vs. 44%, respectively.
	Peterson et al., 2014 [175]	In contrast with the above, a double-blind randomised placebo-controlled trial of ciprofloxacin and probiotic *Escherichia coli* Nissle add-on treatment in 100 patients with active UC found that 78% reached remission in the ciprofloxacin/placebo vs. 89% in the placebo/placebo group.
Rifaximin	Gionchetti et al., 1999 [176]	In the treatment of active UC, rifaximin was found to be better than the placebo.

**Table 9:** The benefits of specific diets in IBD.

Diet	Findings
Specific Carbohydrate Diet	Consuming complex carbohydrates results in a pro-inflammatory microbiome due to fermentation and overgrowth of bacteria when they arrive in the colon [177,178]. Therefore, complex carbohydrates are avoided. Instead, foods that can be eaten include unprocessed meats, most fruits and vegetables, all fats and oils, aged cheeses, and lactose-free yogurt [179]. Following this diet was found to lead to clinical remission in 66% of patients after 10 months, and many were able to stop corticosteroid use [180]. Another survey also demonstrated beneficial results, with 42 % showing remission at both 6 and 12 months [181].
Low FODMAP diet	The low FODMAP diet restricts carbohydrates that are poorly absorbed and highly fermentable [33]. Positive results have been shown when utilising this diet in the management of symptoms relating to irritable bowel syndrome (IBS) [182,183,184]. Yet, there is a lack of knowledge on how underlying inflammation may be affected by this diet [179].
Gluten-free diet	Two large studies have examined its effects. One assessed patients who had a co-diagnosis of coeliac disease, and found that approximately 66% had an improvement in bowel symptoms, and 38% had less severe and frequent IBD flares when on a gluten-free diet [185]. The other study, involving 1254 patients mostly without coeliac disease, found no significant differences between patients following a gluten-free diet and those who were not [186].
Anti-inflammatory Diet	The anti-inflammatory diet (AID) is based on the daily consumption of fruits and vegetables that provide anti-inflammatory compounds such as vitamins B3, B6, E, C, beta-carotene, as well as zinc and magnesium [33]. Olendzki et al., who developed the IBD-AID diet, found that it improved symptoms of patients who were responsive to pharmacological treatment [187].
Mediterranean Diet	The Mediterranean diet involves consuming phytonutrients, replacing saturated and trans-fatty acids with unsaturated fats (such as olive oil), omega-3 polyunsaturated fats, vegetables, high-fibre whole grains, nuts, and a low intake of red meats [33]. In 153 healthy Italian subjects, compliance with a Mediterranean diet resulted in a positive effect on the gut microbiota and associated metabolome [188]. Furthermore, when eight adult patients suffering from CD followed the Mediterranean diet for 6 weeks, their transcriptome analysis showed a change in expression of more than 3000 genes. They also showed that the intestinal microbiota began to normalise [189].

**Table 10:** Studies in non-IBD patients showing the benefit of FVT.

Study	Condition	Findings
Ott et al., 2017 [191]	Recurrent *Clostridioides difficile* infection (rCDI)	Restored normal stool habits of patients and alleviated symptoms of CDI for at least 6 months.
Kao et al., 2019 [192]	Recurrent *Clostridioides difficile* infection (rCDI)	Primary outcome of no recurrence of CDI at the end of 8 weeks post treatment was achieved in 75% (three out of four) patients.
Draper et al., 2020 [193]	Antibiotic-induced dysbiosis	The gut bacteriome was reshaped towards that of pre-antibiotic-treated mice.
Brunse et al., 2022 [194]	Necrotising Enterocolitis	Oro-gastric FVT completely prevented NEC, increased viral diversity, and reduced *Proteobacteria* relative abundance.

**Table 11:** Details of studies looking into the efficacy of phage therapy against *Escherichia coli*.

Study	Findings
Galtier at al., 2017 [202]	Oral treatment with a phage cocktail in colitis mouse model was effective at decreasing colonisation and symptoms over a 2-week period.
Vahedi et al., 2018 [203]	Single dose of phage cocktail was effective at controlling infection.
Yu et al., 2018 [204]	Treatment with phage cocktail was able to control infection, but also led to fewer phage-resistant bacteria.
Febvre et al., 2019 [205]	Taking a commercial cocktail of *E. coli*-targeting phages for 28 days selectively decreased the faecal *E. coli* levels without the gut microbiota community being affected.

**Table 12:** Probiotics used in post-radiotherapy patients.

Probiotic	Effect
*Lactobacillus*	Clinical studies and preclinical models have shown its potential to reduce GI toxicity after RT [209].
	In gynaecological cancer patients who had received pelvic RT, twice daily ingestion >2 × 10^9^ live *Lactobacillus*, resulted in a reduction in diarrhoea symptoms post treatment [210].
*Lactobacillus* and *Bifidobacterium*	Have been found to decrease cancerous tumour size through their influence on immune regulation [207,211].
*L. acidophilus*	Shown to be beneficial against radiation-induced intestinal mucosal injury in rats [212].
*Bifidobacterium*	Reduce chemotherapy-induced mucositis and radiation-induced diarrhoea [213].
*Lactobacillus acidophilus LAC-361* and *Bifidobacterium longum BB-536*	May decrease radiation-induced diarrhoea after the completion of treatment in patients with pelvic cancers [214].
